# Physician’s Attitude Towards Treatment of Older Patients and the Choice of Therapy

**DOI:** 10.4084/MJHID.2013.025

**Published:** 2013-04-10

**Authors:** Felicetto Ferrara

**Affiliations:** Division of Hematology and Stem Cell Transplantation Unit, Cardarelli Hospital, Naples, Italy.

## Abstract

The treatment of acute myeloid leukemia in older patients is still object of controversies, because of considerable heterogeneity among patients and different characteristics in the disease. Reluctance in administering conventional intensive chemotherapy relies on life-threatening complications induced by treatment in an often frail patient population. Nonetheless, while there is general consensus on the management of frail patients with supportive care only, a wide area of uncertainty remains for a considerable proportion of patients in whom treatment beyond support is feasible, with the aim of altering the natural history of the disease. Several predictive score have been proposed in order to prevent toxicity in absence of survival advantage; however in the daily practice patients’ and physician attitude does still play a major role in the final therapeutic decision.

## Introduction

Advanced age represents a common obstacle to cancer patient’s accrual into clinical trials, the aim of which is of establishing new standards of treatment in hematologic and non-hematologic malignancies.^1^ In most cases, exclusion derives from the presence of concomitant diseases and/or age by itself, which render older patients unable to fulfill the inclusion criteria, which often are very stringent.^2^ The inadequate representation in randomized clinical trials of elderly cancer patients leads to a lack of external validity and raises uncertainty regarding benefit-risk ratio of cancer treatment in advanced age.^3^ Notwithstanding, a considerable number of patients judged as ineligible for accrual into trials might receive some form of treatment aimed at altering the natural course of disease. In most cases the choice of type and intensity of such treatments is significantly influenced by patient’s and physician’s attitude and can be further complicated by difficulties in the patient/physician interaction, in that often patients are inclined to avoid detailed information to protect themselves by possible negative emotion.^4^ These aspects are particularly relevant in acute myeloid leukemia (AML), since the most effective therapy we can offer, i.e. intensive induction chemotherapy aiming at complete remission (CR) achievement, is associated with substantial morbidity and mortality, which are not counterbalanced in a considerable proportion of cases by actual survival advantage.^5^,^6^ On the other hand, palliation with best supportive care (BSC) and/or hydroxyurea (HU) to control leukocytosis leads invariably to death and in most cases to repeated hospitalization, because of need of supportive therapy and/or hemorrhagic and infective complications.^7^ Data from Swedish Registry clearly suggest that unselected patients up to the age of 80 years not only can tolerate, but also benefit from intensive treatment, with less early death rates in comparison to palliative approach.^8^ Therefore, with the exception of patients with severe comorbidities and/or those aged over 80 years, a high degree of subjectivity still accounts for the therapeutic choice in a substantial proportion of older patients with AML.^9^ On a theoretical ground, patients should be comprehensively informed about their possibilities of CR achievement and cure, but daily practice suggests that, in most cases, this is easier to say than to be done. In this regard, it should be considered that current therapeutic results in AML diffused across the web or by the media derive from multicenter clinical trials in which relevant selection is operated according to inclusion criteria. As a consequence, overoptimistic expectations are generated and this makes more difficult the patient physician interaction. In a survey undertaken in the United States, 74% of patients estimated their chance of cure to be 50% or greater, whereas 89% of patients the physician anticipated a cure rate of 10% or less.^10^ A clear example of the influence of physician's attitude on therapeutic choice comes from the experience of the Swedish Acute Leukemia Registry. Sweden has six health districts and virtually all patients with AML are registered into a national registry, therefore achieving a full coverage of patients with acute leukemia.^11^ Data clearly show that, while most patients aged 60–69 years were given intensive chemotherapy aiming at CR achievement, the proportion of patients aged 70–79 years considered eligible for CR induction did differ remarkably among the districts, ranging from over 70 % in the North to less than 40 % in the South.^12^ Worthy of note, such a discrepancy resulted in a significantly longer OS in the regions where more elderly AML patients were judged as eligible to intensive chemotherapy. More recently, an analysis of data from the same registry did demonstrate that early death rates were lower in the intensive treatment cohort as compared with the one receiving palliative treatment, suggesting that a substantial proportion of AML patients up to 75 years can tolerate and benefit from intensive induction treatment.^13^ Notwithstanding, in the daily practice a large selection is still made and several factors may account for therapeutic choices other than chemotherapy. As shown in table 1, beside performance status, comorbid diseases and physician and patients' attitude, logistic reasons such as distance from the hematologic institution and presence of a caregiver may guide the therapeutic decision-making process. Finally, a major role is played by the availability for the physician of an innovative clinical trial at the time of diagnosis; in other words, the interest for using new drugs or combination of new with old drugs may induce clinicians to enroll patients in innovative studies, in order to offer a chance to the patient, but also to satisfy their scientific interest.

Current therapeutic option for older patients with AML include intensive chemotherapy followed, whenever possible, by stem cell transplantation procedures, less-intensive chemotherapy, best supportive care and/or hydroxyurea for the control of leukocytosis, and enrollment in clinical trials.^14^,^15^ Difficulties in the therapeutic choice depend on the heterogeneity of patients and disease in advanced age. Ideally, treatment should be tailored on an individual basis, taking into account patients’ and disease’s characteristics, aiming at the achievement of best results with minimal toxicity. It is a common experience that performance status (PS) and age over 75–80 years are still the most significant factors in orienting the final decision. However, in this regard accurate considerations should be made. Not infrequently, apparently frail AML patients improve their PS in a way that they should be re-considered for intensive therapy. Second, since achievement of CR does invariably translate into clinical benefit, due to hematopoietic resumption, good quality of life and no need of supportive therapy for a variable period of time,^16^ even patients aged over 75 years, could be considered for intensive induction, after adequate clinical evaluation. In this specific setting patient/physician interaction has a particular relevance and information about risk/benefit ratio of therapy should be correctly offered. Predictive scores, based on clinical and biologic features have been developed which could help physicians in informing patients.^17^–^19^ However, interaction is complicated by over-optimistic or over nihilistic attitude of patients and relatives, as well as by the formulation of an “acceptable” threshold of death related treatment. Probably, predictive scores are more useful and should be limited to cases with very high probability of death (over 65–70 %), while in the remaining patient population the final decision is still affected by patient’s and physician’s attitude.^9^

More recently, the introduction in the clinical practice of hypomethylating agents Azacytidine (AZA) and decitabine (DAC), has offered further possibility for the management of older patients with AML.^20^ At variance with conventional chemotherapy, both agents can alter the natural course of the disease without necessarily inducing CR and both can be administered on an outpatient basis. This renders AZA and DAC particularly attractive for use in older patients. Currently, AZA has indication for AML with 20–30 % bone marrow blasts and this limits its use to a minority of patients with hypoproliferative AML. AZA causes almost exclusively hematologic toxicity and repeated admissions to hospital are required for 7 days of daily subcutaneous administration. Such aspects are not negligible and may influence therapeutic decision in a substantial proportion of patients. Not surprisingly, analysis of data from AZA compassionate programs as well as from retrospective nationwide surveys produced significantly poorer results as opposed to those of clinical trials, conducted by recruiting highly selected patients. 21–25 On the other hand, DAC has no limitations as to bone marrow blast count it concerns and is specifically licensed for AML patients, in whom conventional intensive chemotherapy is contraindicated. The pivotal study leading to registration by European Medicine Agency (EMA) was based on a randomization between DAC versus either low dose cytarabine (LDARAC) and BSC.^26^ In this study, patients with physician advice were required to preselect, before randomization, the preferred treatment choice (TC) between LDARA-C and BSC. Of interest, a certain degree of imbalance in the number of patients in each treatment arm was observed across participating nations. This was particularly seen in the Western Europe subgroup, which had more patients in the DAC arm (51 patients *vs* 34 patients for TC with physician advice), and in the North American/Australian subgroup, which had more patients in the TC arm (69 patients *vs* 51 patients in the DAC arm), suggesting that patient/physician interaction and their attitude played a major role in the therapeutic decision.

It should be considered that the EMA indication for DAC is for "the treatment of adult patients aged 65 years and above with newly diagnosed de novo or secondary AML, according to the World Health Organization (WHO) classification, who are not candidates for standard induction chemotherapy”. It is also proposed that DAC should be prescribed by physicians experienced in the use of chemotherapeutic agents. Of note, no definite criteria have been suggested to identify patients who are not candidate to intensive induction, leaving the decision to the arbitrary judgment of physician therefore, once again, subjective physician attitude will remain a pivotal driver for the therapeutic decision.

An additional feature requiring consideration concerns the influence on therapeutic decision of the biological findings at diagnosis. In this regard, it is worthy of note that the possibility of achieving CR in patients with unfavorable karyotype (mainly complex or with abnormalities of chromosomes 5 and 7) is lower than 40 %.^5^–^7^ Furthermore, CR in these patients is short-lived and most of them will relapse, despite consolidation chemotherapy, within 6–12 months.^9^ Accordingly, different authors have suggested not to deliver conventional chemotherapy in these AML patients, but rather to offer upon diagnosis, experimental approaches.^27^,^28^ Once again, physician attitude is the driver in that many hematologists are reluctant to endanger the patients’ chance to achieve CR, at least for the age range 60–70. Furthermore, the possibility of subsequent reduced intensity allogeneic stem cell transplantation should not be, a priori, abandoned.^29^ Finally, an experimental therapy can be not immediately available at all institutions managing elderly patients with AML. Notwithstanding, while the treatment of AML patients with unfavorable cytogenetics remains a matter of debate, any effort should be made in order to enroll these patients into clinical trials, using novel drugs.

A final consideration concerns older AML patients relapsed after CR achievement, who represent a major challenge in the daily practice. Physicians' attitude in this setting is extremely variable and range from absolutely nihilistic approaches to excess of treatment. As an example, in a survey conducted and published in 2008 from the Italian Cooperative GIMEMA Group, we showed that the therapeutic approach to relapsed older AML was markedly heterogeneous and only a small minority of patients received experimental therapy.^30^ In this regard, it should be emphasized that relapsed patients with first CR lasting less than 6–12 months as well as those with adverse karyotype at diagnosis will not benefit from conventional salvage treatment, based on high or intermediate dose ARA-C and this information should be carefully pondered and shared with patients and relatives.^31^,^32^

## Conclusions

Although we hope that the next future will witness an ever large representation of biologic predictive parameters for the selection of best therapeutic options in individual patients, for the time being physician's attitude still remains a critical element to direct the therapeutic decision process in elderly patients with AML. In this view, use of simple parameters, rapidly available at bedside, may help restrain judgment subjectivity in defining patients’ unfitness.^33^ This seems particularly appropriate in a time when new drugs are actively developed, so that therapeutic opportunities might be offered even to patients who are ineligible to conventional chemotherapy.^34^ In this way, apart from symptom palliation prolongation of active life expectancy could be offered to a substantial proportion of older patients with AML.^35^ Finally, molecular and genomic prognostic factors are expected to further help in the therapeutic decision.

## Search Strategy and Selection Criteria

As specialists in acute myeloid leukemia, personal knowledge of the relevant literature was the basis for data search and selection criteria in order to support text. References were selected on the basis of their ability to support the text, their appearance in high-impact journals, and recent date of their publication.

## Figures and Tables

**Table 1:** Factors influencing the therapeutic decision in AML of the elderly

Performance status
Comorbidities
Advanced age (>75 years)
Cytogenetic at diagnosis
Secondary AML
Presence of a caregiver
Geographical distance from the hematologic institution
Patients' and relatives' attitude
Physician's attitude and his scientific interest
